# The feasibility of relaxation-enhanced angiography without contrast and triggering for preprocedural planning of transcatheter aortic valve implantation

**DOI:** 10.3389/fcvm.2023.1284743

**Published:** 2023-12-14

**Authors:** Rui Wang, Xinmin Liu, Jing Yao, U. Joseph Schoepf, Joseph Griffith, Jiayang Wang, Jianxiu Lian, Ke Jiang, Guangyuan Song, Lei Xu

**Affiliations:** ^1^ Department of Radiology, Beijing Anzhen Hospital, Capital Medical University, Beijing, China; ^2^ Interventional Center of Valvular Heart Disease, Beijing Anzhen Hospital, Capital Medical University, Beijing, China; ^3^ Division of Cardiovascular Imaging, Department of Radiology and Radiological Science, Charleston, SC, United States; ^4^ Division of Cardiology, Department of Medicine, Medical University of South Carolina, Charleston, SC, United States; ^5^ Center of Coronary Artery Surgery, Department of Cardiac Surgery, Beijing Anzhen Hospital, Capital Medical University, Beijing, China; ^6^ Philips Healthcare, Beijing, China

**Keywords:** transcatheter aortic valve implantation, CT angiography, MRI, cardiac MRI, 3D whole-heart MRI

## Abstract

**Background:**

Cardiovascular MRI is advantageous in transcatheter aortic valve implantation (TAVI) planning. This study aimed to evaluate the feasibility of comprehensive non-contrast MRI [relaxation-enhanced angiography without contrast and triggering (REACT)] combined with a three-dimensional whole-heart MRI protocol for preprocedural planning of TAVI vs. computed tomography angiography (CTA).

**Methods:**

Thirty patients with severe aortic stenosis were prospectively enrolled. The anatomical properties of the aortic root anatomy, including the perimeter and area of the virtual aortic valve annulus and coronary heights, were determined from 3D whole-heart MRI and cardiac CTA (CCTA) images, respectively. The diameters of the aorta (thoracic and abdominal aorta) and iliofemoral arteries were measured from REACT and aortic CTA (ACTA) images, respectively. A paired *t*-test was used to compare these two modalities. Bland–Altman plots were used to assess cardiovascular MRI and CTA measurements. Transcatheter heart valve (THV) sizing was performed based on CCTA measurements and compared with 3D whole-heart MRI measurements. The extent of annular calcification on 3D whole-heart MRI images was evaluated by a four-point grading scale and compared with CCTA data.

**Results:**

All 30 patients completed CTA and cardiovascular MRI examinations, with the TAVI procedure being administered in 25 patients. The mean acquisition time of the comprehensive MRI protocol was 18 ± 3.2 min. There were no significant differences between ACTA and REACT data in regard to the diameters of aortic and iliofemoral arteries, including the ascending thoracic aorta (37 ± 4.6 mm vs. 37.7 ± 5.2 mm, *p* = 0.085), descending thoracic aorta (24.3 ± 2.8 mm vs. 24.3 ± 2.8 mm, *p* = 0.832), abdominal aorta (20.9 ± 2.5 mm vs. 20.8 ± 2.5 mm, *p* = 0.602), bilateral common iliac arteries (right: 8.36 ± 1.44 mm vs. 8.42 ± 1.27 mm, *p* = 0.590; left: 8.61 ± 1.71 mm vs. 8.86 ± 1.46 mm, *p* = 0.050), and bilateral femoral arteries (right: 6.77 ± 1.06 mm vs. 6.87 ± 1.00 mm, *p* = 0.157; left: 6.75 ± 1.02 mm vs. 6.90 ± 0.80 mm, *p* = 0.142). Both modalities showed similar aortic valve morphology and semi-quantitative valve calcification (all, *p*'s > 0.05). Overall agreement for implanted THV was found in all 25 (100%) patients assessed with both modalities.

**Conclusion:**

REACT combined with 3D whole-heart MRI enables reliable measurements of aortic root anatomy, annular calcification, and aorta and iliofemoral access in patients under evaluation for TAVI.

## 1. Introduction

Transcatheter aortic valve implantation (TAVI) has emerged as a viable treatment strategy for non-surgical cases with symptomatic severe aortic stenosis (AS), from high- to low-risk cases (1–3). Computed tomography angiography (CTA) is considered the standard imaging method for pre-interventional evaluation and planning of TAVI, including annular sizing, determining the risk of annular injury and coronary occlusion, evaluating the coplanar fluoroscopic angle, and assessing peripheral access (4, 5). However, a notable disadvantage of CTA is the requirement for a large volume of a contrast agent, which increases the odds of contrast-induced nephropathy (CIN) and acute renal failure (6). Unfortunately, most TAVI patients (up to 80%) are septuagenarians and octogenarians with chronic renal impairment (7). Thus, a non-contrast high-definition imaging tool is particularly crucial for the preprocedural planning of TAVI.

Cardiovascular magnetic resonance imaging (CMRI) using techniques for non-contrast 3D MR angiography (MRA), including 3D whole-heart MRI, has shown great potential in TAVI planning (8–10), including radiation- and contrast-free assessment of aortic annular sizing (8–10). Furthermore, CMR enables the evaluation of aortic valve stenosis and regurgitation and ventricular function (11, 12). However, the main limitation of non-contrast MRA is the inability to depict the femoral access or the whole aorta for TAVI planning (12). CE-MRA for TAVI guidance is available (13) but requires contrast agent injection.

Recently, a novel 3D relaxation-enhanced angiography without contrast and triggering (REACT) sequence was developed (14, 15), which combines non-volume-selective short inversion recovery (STIR) and T2 preparation pulses with dual-gradient echo Dixon (mDIXON) readout. The latter technique utilizes the advantages of balanced steady-state free precession (bSSFP), including bright-blood signals, with robust fat and background suppression for flow-independent isotropic 3D non-contrast MRA (15). REACT provides a simultaneous assessment of arterial and venous vessels, with encouraging results in displaying pulmonary vasculature in congenital heart disease (14, 16), extracranial internal carotid artery (17) and thoracic aorta (18, 19), and pelvic venous vessels (16). However, it is unclear whether REACT can accurately measure the whole aorta, including the common iliac artery, external iliac artery, and femoral artery in TAVI patients.

The purpose of this study was to evaluate a non-contrast CMR protocol comprising 3D whole-heart MRI for the assessment of aortic root anatomy and REACT for the depiction of the aorta and iliofemoral arteries for preprocedural analysis of TAVI patients.

## 2. Materials and methods

### 2.1. Patient population

This prospective study was performed at a single hospital center and approved by the Institutional Review Board (Approval No. 2021061X). Signed informed consent was obtained from all patients. Thirty patients with severe symptomatic aortic stenosis referred for ECG-gated cardiac CTA and cardiovascular MRI examinations for potential TAVI were enrolled between August 2021 and June 2022. The New York Heart Association (NYHA) functional classification was employed to categorize LV function (NYHA II–IV) (20). Preprocedural Society of Thoracic Surgeons (STS) scores were obtained with a freely available online calculator (http://riskcalc.sts.org/stswebriskcalc/#/). High surgical risk was defined as an STS score ≥8%, and low or intermediate risk was defined as an STS score <8% (1).

Exclusion criteria were severe renal failure (glomerular filtration rate GFR <30 ml/min), atrial fibrillation, unstable angina pectoris, or acute myocardial infarction within 30 days before enrollment, previous valve surgery, intracardiac shunt, previous reaction to iodinated contrast agents, a permanent pacemaker, a ferromagnetic foreign body, and known contraindications for MRI imaging.

### 2.2. CTA protocol

All patients were examined on a 256-slice CT system (Revolution CT, GE Healthcare) with a gantry rotation time of 0.28 s/rotation, a tube voltage of 100 kV, and a tube current range of 120–570 mA. The comprehensive CTA protocol comprised a retrospective ECG-gated cardiac computed tomographic angiography (CCTA) examination followed by a routine non-ECG-gated aortofemoral CTA (ACTA) examination. Retrospective ECG-gated CCTA was employed for whole cardiac cycle acquisition (0%–100% of the R-wave). The scan range of the ACTA extended from subclavian arteries to the femoral arteries with a helical, non-ECG-gated data acquisition delay of 2 s following CCTA.

In total, 50–70 ml of intravenous contrast agent (iomeprol 350 mg/ml, Beilu, Beijing, China) was injected at a flow rate of 4 ml/s, followed by saline injection (30 ml) at the same flow rate through an 18-G needle into an antecubital vein. A bolus tracking algorithm automatically started the scan 3 s after reaching a prespecified threshold of 100 Hounsfield units (HU) in the ascending aorta.

CCTA images were reconstructed with a section thickness of 1 mm and no increment at 5% steps throughout the cardiac cycle. These settings were selected because end-systole [30%–40% Rwave - R wave (RR) interval] is the recommended phase for assessing the aortic annulus during TAVI planning. ACTA images were reconstructed with a section thickness of 1 mm and no increment. All datasets were transferred to a dedicated postprocessing workstation.

### 2.3. MRI protocol

All scans were performed on a 3 T MRI system (Philips Ingenia CX, Philips Healthcare, Best, Netherlands) equipped with a standard 32-channel coil.

### 2.4. 3D whole-heart MRI protocol

A free-breathing, non-contrast, Dixon water-fat separation 3D whole-heart MRI sequence was applied (21), with the following settings: time to echo (TE), 1.54 ms; repetition time (TR), 3.5 ms; flip angle, 10^°^; compressed sensing (CS) factor, 2.5; k-space Cartesian; matrix, 256 × 184 × 250 mm; mDIXON, ECG-gated; the trigger delay, 240–300 ms of the temporal window. This acquisition window was determined by a four-chamber view of the left ventricle with reduced motion and a turbo field echo (TFE) shot duration of 80–130 ms, depending on the heart rate. The gating window of the navigator respiratory compensation (with pencil beam) was 6 mm. Reconstruction parameters are as follows: real time; spatial resolution, 1.5 mm × 1.5 mm × 1.5 mm; gap, −0.75 mm. The scan time of 3D whole-heart MRI was 13 ± 1.5 min.

### 2.5. REACT sequence

Aortic imaging was based on a flow-independent 3D isotropic REACT sequence combining a 50-ms T2 preparation sequence and a STIR pulse with a 3D mDIXON XD readout (22). Imaging parameters for the REACT sequence included the following: scan mode, 3D fast field echo (FFE); acquisition mode, Cartesian; acquired resolution, 2 mm × 2 mm × 3 mm; reconstructed resolution, 1.3 mm × 1.3 mm × 1.5 mm; field of view, 350 mm × 394 mm × 120 mm; matrix, 152 × 165 × 80; TR/TE, shortest; compressed SENSE, 3; no navigator; and ECG-gated. Because the entire aorta was included, there were three slabs with 20% overlap from the subclavian arteries to the bilateral femoral arteries. The scan time was 2 min per slab, for a total scan time for the REACT sequence of 6 min.

### 2.6. Analysis of CTA and cardiovascular MRI

CTA images were analyzed by two interventional cardiologists (XL, 15 years as a cardiologist, and JY, 10 years as an interventional cardiologist) with 3mensional structural heart software 10.3 (Maastricht, Netherlands), which enables free navigation and image plane selection of the 3D dataset. CMR images were analyzed by a radiologist (RW, 13 years as a cardiovascular radiologist) using a dedicated Philips IntelliSpace Portal workstation (version 10.1, Philips Healthcare, Best, Netherlands). The following methods were used to assess quantitative parameters.

Aortic root anatomy: As previously described (5), the aortic annulus (area, perimeter, and diameter) at the hinge point plane, left ventricular outflow tract (LVOT), and the coronary ostium heights were measured by CCTA and 3D whole-heart MRI.

Morphology and calcification of the aortic valve: The morphology of the aortic valve was categorized as bicuspid or tricuspid, as assessed from CCTA and 3D whole-heart MRI data. Furthermore, a bicuspid aortic valve (BAV) was classified into three subcategories (23): (1) fused BAV, (2) 2-sinus BAV, and (3) partial-fusion BAV. The extent of calcification of the aortic annulus was analyzed by a semi-quantitative method for 3D whole-heart MRI and CCTA with a four-point scale (24): (1) mild calcification, (2) moderate calcification, (3) heavy calcification (mostly involving any each of commissural fusion), and (4) calcification protruding into the LVOT.

Diameters of the aorta and iliofemoral arteries (11): The diameters of the ascending and descending thoracic aortas, abdominal aorta, and bilateral iliofemoral arteries were measured by REACT and ACTA. In this study, the diameter of the ascending thoracic aorta was measured at the level up to 40 mm from the aortic valve, the descending thoracic aorta was measured at the level of trachea bifurcation, and the abdominal aorta was measured at the level of the celiac track artery. The minimum diameters of common iliac arteries and external iliac arteries were measured, and femoral arteries were measured at the proximal segment.

### 2.7. Hypothetical transcatheter heart valve sizing

Hypothetical prosthesis sizing was selected based on systolic CCTA and 3D whole-heart MRI measurements following the manufacturer's instructions. Systolic CCTA was considered the gold standard for transcatheter heart valve (THV) selection. For self- or balloon-expandable valves, the effective diameter was extrapolated from the annular perimeter or the annular area. The perimeter-derived effective diameter was determined as perimeter/π, while the area-derived effective diameter was assessed as $2\times \sqrt{(\mathrm{area}/\pi }$ (25). The selected THV was considered the borderline size, allowing for the choice of the adjacent prosthesis size. Selection of the identical sizing category was considered “exact agreement,” and discrepancy levels of 1 and ≥2 sizing categories were considered “extended agreement” and “disagreement,” respectively (9).

### 2.8. Statistical analysis

Statistical analysis was performed using SPSS version 22.0 (SPSS, Chicago, IL, USA) and MedCalc version 15.8. Continuous variables with normal distribution were reported as mean ± standard deviation. Categorical variables were compared by the chi-squared test. Categorical data were reported as frequencies and percentages. Paired Student's *t*-test was used to compare aortic root and whole aorta features obtained from cardiovascular MRI and CTA data. For linear correlation analysis, the Spearman rank correlation coefficient (*ρ*) was calculated. To assess the degree of agreement between cardiovascular MRI and CTA data for each pair of aortic roots, whole aorta, bilateral common iliac arteries, bilateral external iliac arteries and bilateral femoral arteries, Bland–Altman plots including mean differences and limits of agreement were obtained. *p* < 0.05 was considered statistically significant.

## 3. Results

### 3.1. Patients’ characteristics

**Figure 1:**
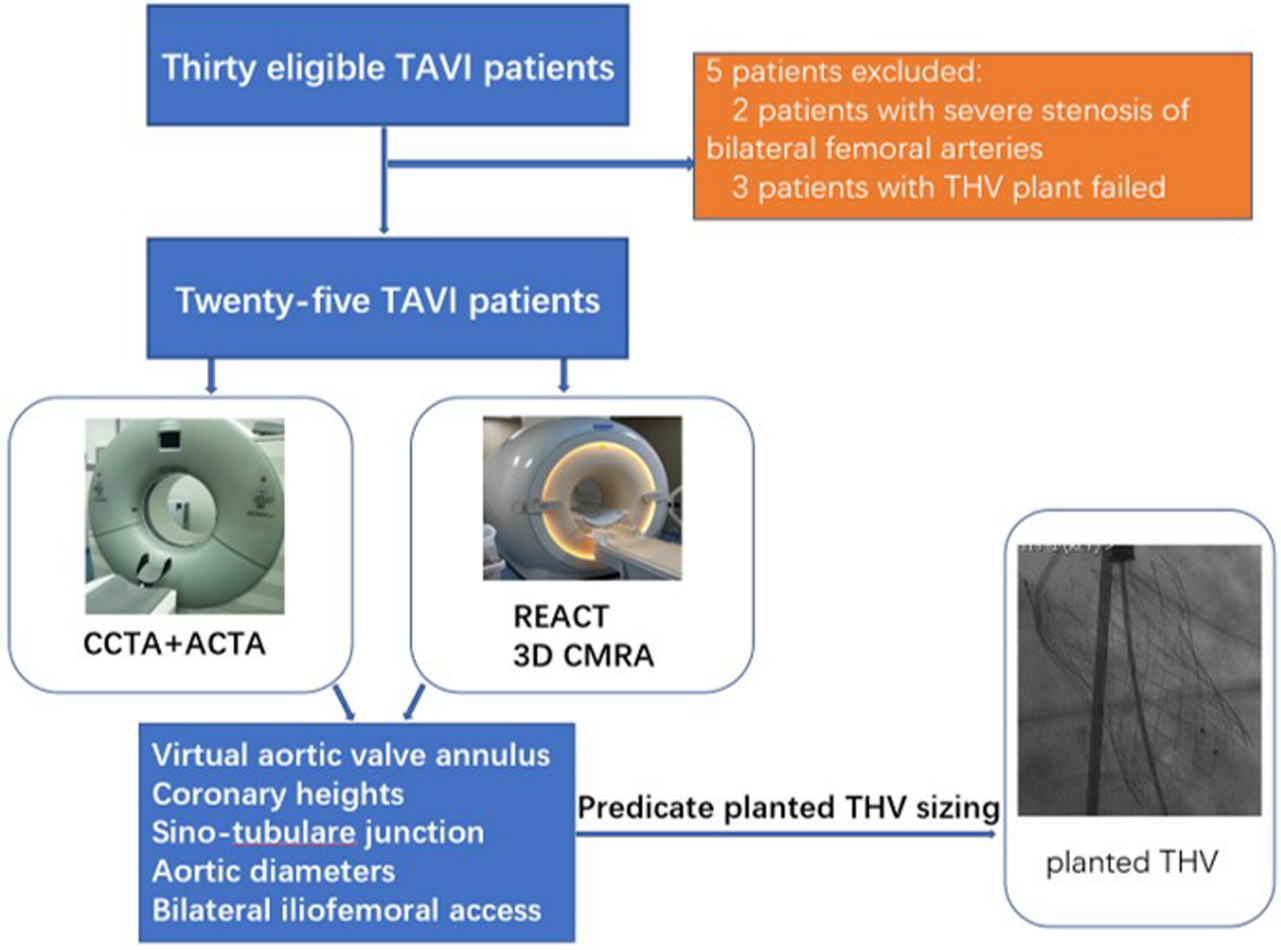
Study workflow.

CTA and cardiovascular MRI examinations were successfully completed for all 30 patients. Of these, 25 patients successfully underwent TAVI (14 women, average age: 68 ± 7 years), including 23 and two who received self-expandable THVs and balloon-expandable THVs, respectively. Five patients were excluded, including two with severe stenosis of bilateral femoral arteries and three with failed THV implantation. The study workflow is shown in Figure 1. All patients had no allergic reaction to the CTA contrast agent. Patients’ characteristics are summarized in Table 1.

**Table 1:** Baseline patient characteristics.

Variable	
Age (year)	68 ± 7
Gender	16 males/14 females
BMI (kg/m^2^)	24 ± 3
Heart failure with NYHA	NYHA I 0 (0%)
	NYHA II 8 (27%)
	NYHA III 18 (60%)
	NYHA IV 4 (13%)
Echocardiography—LVEF %	>50%—2 (7%)
	40%–49%—22 (73%)
	<40% 6 (20%)
Aortic valve area (cm^2^)	0.67 ± 0.12
STS score (%)	5.2 ± 2.1

BMI, body mass index.

### 3.2. Aortic root and whole aorta dimensions

**Figure 2:**
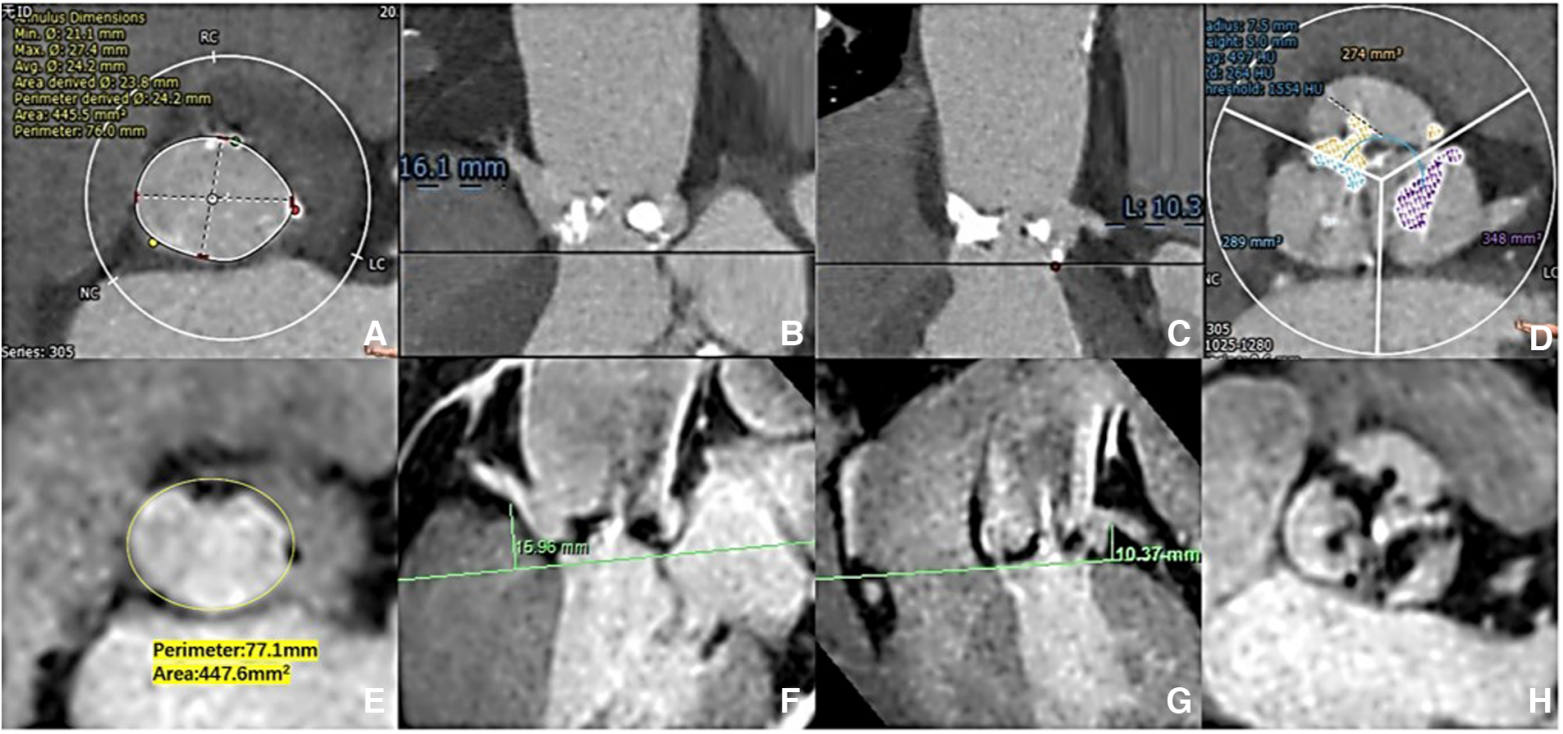
Aortic root measurement: CCTA vs. 3D whole-heart MRI. The aortic ring dimensions were assessed [(**A**) CCTA vs. (**E**) 3D whole-heart MRI]. The right/left coronary ostial heights were measured perpendicular to the annulus plane [(**B**,**C**) CCTA: vs. (**F**,**G**) 3D whole-heart MRI]. The extent of valve calcification was assessed [(**D**) CCTA vs. (**H**) 3D whole-heart MRI).

**Figure 3:**
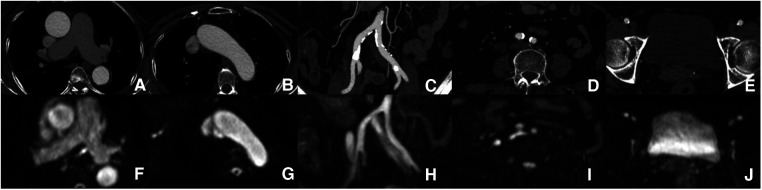
Aorta and iliofemoral access measurements. Each part of the aorta and iliofemoral arteries were measured by both modalities [(**A**–**E**) ACTA vs. (**F**–**J**) REACT].

**Figure 4:**
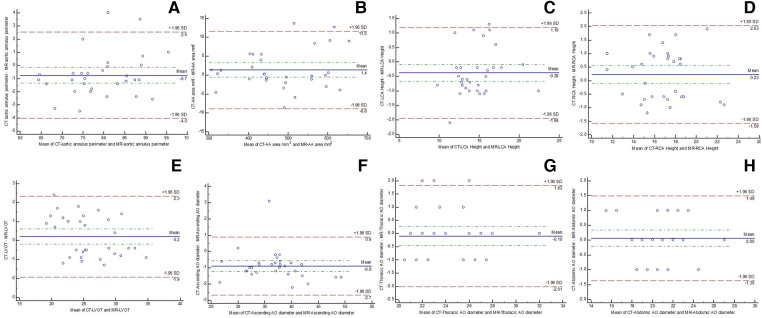
Bland–Altman plots showing the agreement between CTA and comprehensive MRI in measuring aortic root [(**A**) annular perimeter; (**B**) annular area], bilateral coronary heights [(**C**) left; (**D**) right], (**E**) LVOT, (**F**) ascending thoracic aorta, (**G**) descending thoracic aorta, and (**H**) abdominal aorta.

No significant differences were found in the perimeter, area, and average diameters of the annular ring and in coronary ostial heights between 3D whole-heart MRI and CCTA data. The diameters of the ascending thoracic aorta (37 ± 4.6 mm vs. 37.7 ± 5.2 mm, *p* = 0.085), descending thoracic aorta (24.3 ± 2.8 mm vs. 24.3 ± 2.8 mm, *p* = 0.832), abdominal aorta (20.9 ± 2.5 mm vs. 20.8 ± 2.5 mm, *p* = 0.602), bilateral common iliac arteries (right: 8.36 ± 1.44 mm vs. 8.42 ± 1.27 mm, *p* = 0.590; left: 8.61 ± 1.71 mm vs. 8.86 ± 1.46 mm, *p* = 0.050), and bilateral femoral arteries (right: 6.77 ± 1.06 mm vs. 6.87 ± 1.00 mm, *p* = 0.157; left: 6.75 ± 1.02 mm vs. 6.90 ± 0.80 mm, *p* = 0.142) showed no significant differences between REACT and ACTA data. Excellent agreement was found in aortic root dimensions and aorta and iliofemoral access route measurements for both modalities (Tables 2,3, Figures 2–4).

**Table 2:** MRI and CTA measurements between both modalities and correlation coefficients.

	CTA	MRI	*t*	*p*-value
Aortic annular perimeter (mm)	79.6 ± 9.4	80.3 ± 9.0	1.957	0.061
Aortic annular area (mm^2^)	489.2 ± 108.7	487.8 ± 108.3	−1.109	0.278
Left coronary height (mm)	14.8 ± 3.2	15.2 ± 3.0	2.705	0.052
Right coronary height (mm)	17.2 ± 2.9	16.9 ± 2.9	−1.497	0.147
LVOT diameter (mm)	26.1 ± 4.1	25.9 ± 4.3	−1.133	0.267
Ascending thoracic aorta (mm)	37 ± 4.6	37.7 ± 5.2	1.792	0.085
Descending thoracic aorta (mm)	24.3 ± 2.8	24.3 ± 2.8	0.214	0.832
Abdominal aorta (mm)	20.9 ± 2.5	20.8 ± 2.5	−0.527	0.602
Right common iliac artery (mm)	8.36 ± 1.44	8.42 ± 1.27	0.545	0.590
Left common iliac artery (mm)	8.61 ± 1.71	8.86 ± 1.46	2.044	0.05
Right external iliac artery (mm)	6.56 ± 1.09	6.67 ± 1.06	1.849	0.079
Left external iliac artery (mm)	6.81 ± 1.11	7.00 ± 1.10	1.156	0.360
Right femoral artery (mm)	6.77 ± 1.06	6.87 ± 1.00	1.451	0.157
Left femoral artery (mm)	6.75 ± 1.02	6.90 ± 0.80	1.510	0.142

**Table 3:** Correlation between comprehensive MRI and CTA measurements.

Comparison of CTA vs. MRI	*ρ*-value*	Bland–Altman
Aortic annular perimeter	0.982	−0.7 (−4.0/2.5)
Aortic annular area	0.998	1.4 (−8.8/11.6)
Left coronary height	0.967	−0.38 (−1.94/1.19)
Right coronary height	0.951	0.23 (−1.58/2.03)
LVOT diameter	0.964	0.2 (−1.9/2.3)
Ascending aorta	0.918	0.9 (−2.7/0.9)
Thoracic aorta	0.947	−0.10 (2.01/1.82)
Abdominal aorta	0.956	0.06 (−1.36/1.49)
Right common iliac artery	0.890	−0.06 (−1.36/1.23)
Left common iliac artery	0.918	−0.25 (−1.6/1.09)
Right external iliac artery	0.981	−0.28 (−0.92/0.37)
Left external iliac artery	0.952	−0.51 (−1.54/0.52)
Right femoral artery	0.928	−0.24 (−1.21/0.73)
Left femoral artery	0.824	−0.29 (−1.7/1.12)

* *p* < 0.0001.

### 3.3. Morphology of the aortic valve and annular calcification

The morphology of the aortic valve and annular calcification were analyzed for both 3D whole-heart MRI and CCTA images. No significant differences were found in aortic valve morphology and subjective scores for aortic valve calcification for both modalities (Table 4).

**Table 4:** Morphology of the aortic valve and annular calcifications scores obtained by 3D whole-heart MRI and CCTA.

	CCTA	3D whole-heart MRI
Morphology of the aortic valve		
Tricuspid	14	14
Bicuspid	16	16
Fused BAV	10	10
2-sinus BAV	6	6
Partial-fusion BAV	0	0
Aortic valve calcification		
Score 1	7	9
Score 2	11	10
Score 3	11	10
Score 4	1	1

### 3.4. THV size

Based on CCTA annulus measurements, decisions for prosthesis size in preprocedural planning were concordant in all individuals. THV sizing showed exact agreement for 3D whole-heart MRI with implanted THV in 14 of 25 patients (56%) and for CCTA with implanted THV in 12 of 25 patients (48%). The overall agreement (exact and extended agreement) in implanted THV was 100% (25/25) for both modalities (3D whole-heart MRI and CCTA) (Table 5).

**Table 5:** Predicted valve sizes according to 3D whole-heart MRI and CCTA measurements vs. size of actual implanted valves.

Self-expandable valves		Implanted THV			
		23 mm	26 mm	29 mm	32 mm
3D whole-heart MRI	23 mm	5	1		
	26 mm	3	4		
	29 mm		3	2	2
	32 mm			2	1
CCTA	23 mm	4	1		
	26 mm	4	3		
	29 mm		4	1	1
	32 mm			3	2
Balloon-expandable valves		Implanted THV			
		21 mm	23 mm	26 mm	29 mm
3D whole-heart MRI	21 mm	1			
	23 mm				
	26 mm			1	
	29 mm				
CCTA	21 mm	1			
	23 mm				
	26 mm			1	
	29 mm				

## 4. Discussion

This study assessed a comprehensive non-contrast cardiovascular MRI protocol (combining REACT and 3D whole-heart MRI) for imaging the aortic root and the whole aorta and its branches for preprocedural assessment of TAVI patients. The results of this study indicate that REACT is feasible for route evaluation of TAVI patients while providing excellent agreement to CTA. The combination with 3D whole-heart MRI enables contrast-free preoperative assessment in a clinically feasible scan time.

REACT, a novel non-contrast MRA method, can provide an isotropic 3D readout enabling image reconstruction in all three dimensions (15). It was demonstrated that REACT reliably measures thoracic aorta dimensions (18, 19, 26) and pulmonary vasculature (14) with high intra- and inter-observer agreements. Moreover, for clinically relevant carotid artery stenosis (≥50%), REACT achieved a detection sensitivity of 93.75% and a specificity of 100% (27). As shown above, the diameters of the whole aorta (ascending, thoracic, and abdominal aortas), bilateral common iliac arteries, bilateral external iliac arteries, and bilateral common femoral arteries showed no significant differences between REACT and CTA. Pennig et al. and Isaak et al. found an average acquisition time of 6 min in REACT for thoracic vasculature (18, 26). However, three imaging slabs (overlapping 20% of each slab) were used to cover from the carotid artery to bilateral femoral arteries; each slab required an acquisition time of 2 min, making a total scan time of 6 min in this study, corroborating a previous study. There were two reasons: (1) ECG-trigger and respiratory navigator-trigger were not used; (2) compared to previous studies on the thoracic vasculature (18, 26), a relatively inferior resolution (2 mm^3^ × 2 mm^3^ × 3 mm^3^) of the sequence was applied, which resulted in shorter acquisition time.

In addition to REACT, quiescent interval slice-selective (QISS)-MRA is another non-contrast MRA technique, which uses flow-compensated fast low-angle shot readout and exhibits promising results for visualizing diverse vascular territories, including the carotid artery (28), coronary arteries (29), pulmonary arteries (30), abdominal and pelvic arteries (31), and the peripheral artery (32), without a contrast agent. However, there are potential drawbacks because of 2D acquisition of QISS-MRA depends on the inflow of spins from outside the saturation volume, which is associated with technical limitations, including anisotropic image volumes, long acquisition time, blood flow dependency, and vessel orientation relative to imaging slices. Thus, if the whole aorta anatomy was imaged by the QISS-MRA sequence, the aortic arch must be scanned twice to visualize the ascending and descending aortic segments separately because the QISS-MRA sequence requires tracking pre-saturation pulse on opposite sides, respectively (33). In this study, the REACT sequence is a 3D acquisition protocol without limitation of blood flow orientation. However, a shortcoming of REACT is that venous signals were not well suppressed. Pamminger's results demonstrated that the non-contrast QISS-MRA can evaluate aortoiliofemoral vessel diameters in TAVI patients in around 9 min (33).

Renker et al. assessed the feasibility of a novel, non-contrast, free-breathing, self-navigated three-dimensional MRA protocol (10). However, the latter MRA protocol was not used for examining TAVI candidates. CE-MRA is equivalent to CTA in TAVI preplanning (13). Mayr et al. showed that vessel luminal diameters and the angulations of aortofemoral access as measured by CE-MRA and CTA showed overall robust correlations (*r* = 0.819–0.996, all *p* < 0.001), and agreement analysis for minimal vessel diameter between both modalities showed a bias of 0.02 mm (13). Unfortunately, administration of gadolinium-based contrast agents in patients with severely impaired renal function is contraindicated because of the risk of nephrogenic systemic fibrosis (NSF) (34).

It has also been demonstrated that 3D whole-heart MRI allows for a more reliable assessment of aortic annulus dimensions and the extent of calcification, even in the presence of arrhythmias, in the pre-TAVI population compared with CCTA (8, 9). In a study by Ruile et al., 3D whole-heart MRI in the systolic phase was modeled using a corrective factor of the relative difference between systolic and diastolic CTA area dimensions since the trigger delay was set during diastole (9). Here, the trigger delay was 240–300 ms of the temporal window in the 3D whole-heart MRI sequence, which was similar to the systolic phase of the RR interval; thus, the measurements of the aortic annular perimeter and area showed no significant difference between CCTA and 3D whole-heart MRI data. The development of non-contrast 3D whole-heart MRI has progressed rapidly in recent years. Pamminger et al. showed that self-navigated whole-heart MRI produces aortic annulus TAVI measurements without significant differences vs. navigator-gated whole-heart MRI. The benefit of self-navigated whole-heart MRI was short acquisition time (5.5 ± 1 min vs. 6.5 ± 2 min, *p* = 0.029) (35). In this study, acquisition time was 10–15 min using an ECG and a navigator-gated whole-heart MRI protocol, depending on the patient's heart rate and breathing pattern. It is difficult to quantitatively assess aortic valve calcification (e.g., CT calcium score) by traditional MRI (4). However, 3D whole-heart MRI offers high isotropic resolution, enabling easy reformatting of the dataset in a given orientation. The current results showed that semi-quantitation of annular calcification from 3D whole-heart MRI images using a four-point grading scale showed no statistically significant difference compared to CCTA quantification, corroborating Ruile et al. (9).

The present study had several limitations. First, the sample size was relatively small and more patients referred for TAVI evaluation should be enrolled in future studies. Second, valve annular calcifications cannot be quantified by MRI; therefore, semi-quantitation of annular calcification based on 3D whole-heart MRI was performed. Finally, the scan time was relatively longer for the comprehensive non-contrast MRI protocol (averaging 18 min) than for CTA. Moreover, a compressed SENSE factor of 3 was used in this study and different CS factors should be evaluated in further studies.

## 5. Conclusion

In conclusion, REACT combined with 3D whole-heart MRI is suitable for preprocedural evaluation of TAVI patients without requiring an iodinated contrast agent or ionized radiation.

## Data Availability

Restrictions apply regarding the use of CTA and MRI data used in this study. The raw data supporting the conclusions of this article will be made available by the authors where permissible.
